# Exploring the gut microbiota-Parkinson’s disease link: preliminary insights from metagenomics and Mendelian randomization

**DOI:** 10.3389/fmicb.2025.1654418

**Published:** 2025-09-26

**Authors:** Jiaji Liu, Le Wang, Ling Su, Jiayi Chen, Ruijun Su

**Affiliations:** ^1^Inner Mongolia Medical University, Department of Laboratory Medicine, Affiliated Hospital of Inner Mongolia Medical University, Hohhot, China; ^2^Department of Laboratory Medicine, Hohhot First Hospital, Affiliated Fifth Clinical Medical College of Inner Mongolia Medical University, Hohhot, China

**Keywords:** metagenomics, Mendelian randomization, microbiome, gut, Parkinson’s disease, short-chain fatty acids

## Abstract

**Introduction:**

The relationship between the gut microbiome and Parkinson’s disease (PD) has recently attracted significant attention, with most studies focused on analyzing microbial composition. However, our understanding of the potential causal relationship between the gut microbiota and PD remains limited.

**Methods:**

We extracted microbiome data from the metagenome for broad taxonomic coverage and accurate functional analysis. Subsequently, Mendelian randomization was employed to elucidate the causal relationship between the gut microbiome and PD.

**Results:**

The gut microbiota in PD patients was found to be systemically imbalanced, characterized by an abnormal enrichment of potential pathogenic bacteria, a significant reduction in key beneficial bacteria, and a reorganization of intestinal metabolic functions. This state of imbalance involves significant abnormalities in multiple metabolic and regulatory pathways, including the glucose metabolism, oxidative stress response, protein homeostasis regulation, and immune signaling pathways. These findings suggest that dysbiosis may influence host neural function through multilevel metabolic interventions. Additionally, specific microbial communities are clearly associated with disease risk, with some bacterial populations promoting disease onset and others demonstrating a potentially protective effect. Although metagenomic findings require validation in larger cohorts, the results of this study indicate that changes in gut microbiota composition and function are closely related to PD onset and progression.

**Conclusion:**

This study revealed that certain microorganisms traditionally considered beneficial may contribute to PD risk. This finding challenges previous assumptions and highlights the complexity of host–microbiome interactions. The identification of altered metabolic and immune pathways, particularly those involving bacteria that produce short-chain fatty acids, underscores the critical role of the gut microbiota in PD pathophysiology. However, the relatively small sample size of the current metagenomic analysis limits the generalizability of these findings. Larger, more diverse cohorts are needed to validate these results. Despite this limitation, the study provides important insights into microbiome-targeted therapeutic strategies, emphasizing the need to reconsider the roles of both beneficial and harmful microorganisms in PD.

## 1 Introduction

According to the 2019 Global Burden of Disease Study, the absolute numbers of deaths and disability-adjusted life years (DALYs) attributable to Parkinson’s disease (PD) have increased significantly in recent years, driven by global population aging and the improved survival rates of middle-aged and older adults (GBD 2016 Neurology Collaborators, 2019; Han et al., 2025). Consequently, PD has emerged as a substantial global health burden that cannot be ignored. In terms of clinical manifestations, PD is characterized by progressive motor and cognitive impairments and gastrointestinal dysfunctions, such as constipation and delayed gastric emptying; these gastrointestinal symptoms often precede the onset of motor symptoms by several years (Váradi, 2020). The early onset of these nonmotor symptoms has increasingly drawn researchers’ attention to the underlying mechanisms linking the gut and the central nervous system (CNS). Thus, an increasing number of research studies focus on the concept of the “gut–brain axis” (GBA) and the specific role of the gut microbiota in PD pathogenesis (Cryan et al., 2019). Gut microbiota dysbiosis has been identified as a significant exogenous contributor to the pathogenesis of PD. Emerging evidence indicates that specific bacterial taxa can activate innate immune pathways, particularly through Toll-like receptors (TLR2 and TLR4), thereby promoting the abnormal aggregation of *α*-synuclein (α-Syn) within the enteric nervous system (Gorecki et al., 2021). As a pivotal pathogenic protein in PD, misfolded α-Syn constitutes the core component of Lewy bodies and is capable of propagating from the gut to the central nervous system (CNS) via the vagus nerve, forming a pathological continuum that supports the gut-origin hypothesis of PD (Braak et al., 2006). Concurrently, dysfunction of the GBA compromises intestinal barrier integrity, increasing permeability and facilitating the translocation of microbial metabolites and pro-inflammatory molecules, such as lipopolysaccharide (LPS), into the systemic circulation (Klann et al., 2021). These circulating factors can activate microglia and trigger chronic neuroinflammation. Clinical studies have demonstrated significantly elevated levels of inflammatory cytokines, including tumor necrosis factor-*α* (TNF-α), interleukin-6 (IL-6), and interleukin-1β (IL-1β), in both intestinal and brain tissues of PD patients, underscoring the contribution of systemic inflammation to disease progression (Verma et al., 2025). Moreover, the depletion of beneficial microbial metabolites, particularly short-chain fatty acids (SCFAs), exacerbates inflammatory responses and may enhance the translocation of *α*-Syn across the GBA (Fitzgerald et al., 2019; Mahbub et al., 2024). Certain bacterial components, such as curli fimbriae proteins and LPS, have been shown to directly interact with α-Syn, accelerating its aggregation and promoting oxidative stress (Chen et al., 2016; Taglialegna et al., 2016). Additionally, sulfate-reducing bacteria, including members of the genus *Desulfovibrio*, can produce neurotoxic metabolites such as hydrogen sulfide, which may further impair neuronal function and exacerbate neurodegeneration through direct toxic mechanisms (Alam et al., 2024).

It is essential to elucidate the gut microbiome–PD association more comprehensively to identify relevant information about the microbial sources of risk associated with this disease. The advent of metagenomic techniques has provided a refined and comprehensive approach to such investigations (Villette et al., 2025). Conventional 16S rRNA gene amplicon sequencing enables the analysis of overall gut microbial community structure; however, as this approach is generally limited to genus-level resolution, it cannot readily distinguish closely related taxa and is insufficient for in-depth functional characterization (Papić et al., 2025). In contrast, metagenomics, which involves sequencing the entire microbial genome, enables the discrimination of microbes at the species or even strain level, permitting the accurate elucidation of functional differences, such as those involved in carbohydrate metabolism, protein degradation, and SCFA synthesis (Wallen et al., 2022; Boktor et al., 2023). The precision and comprehensiveness of metagenomics provide more reliable data for understanding the role of the gut microbiota in complex neurodegenerative diseases involving multifactorial interactions and complicated disease progression, such as PD.

However, most existing studies have been observational, lacking robust evidence of causality. It remains unclear whether alterations in the gut microbiota of patients with PD act as a driving factor in disease onset or represent a secondary effect that emerges during disease progression. To address this limitation, Mendelian randomization (MR) has been increasingly applied in studies investigating the relationship between the gut microbiota and diseases (Jiang et al., 2023). MR utilizes single nucleotide polymorphisms (SNPs) associated with specific microbiota traits, effectively simulating a “natural randomized controlled trial” in populations, thereby better controlling for environmental and other confounding factors (Sekula et al., 2016). MR can significantly reduce bias in analyses of complex diseases such as PD, thereby enabling a more precise identification of the mechanisms by which the gut microbiota contributes to disease progression.

This study was conducted to systematically investigate the gastrointestinal symptoms commonly observed in the early stages of PD and their potential associations with the GBA, leveraging the high-resolution and functional profiling capabilities of metagenomics. The widespread presence of gastrointestinal symptoms among early-stage PD patients underscores the value of in-depth exploration of the GBA. Metagenomic technologies provide comprehensive analytical support, enabling more precise elucidation of the relationship between the gut microbiota and PD progression. Moreover, the application of MR offers a robust tool for causal inference, effectively minimizing the influence of confounding factors and reverse causality. While our results offer valuable insights, they should be regarded as hypothesis-generating, and further validation in diverse. As longitudinal and large-scale cohort studies continue to advance, we anticipate a clearer understanding of the specific role of the gut microbiota in PD pathogenesis, paving the way for novel approaches to clinical intervention and personalized treatment strategies.

## 2 Methods

### 2.1 Metagenomics

#### 2.1.1 Study population and sample collection

A case–control study was conducted from January 2021 to June 2022 involving 25 patients (13 males, 12 females) diagnosed with early-stage PD at the Neurology Department of the Affiliated Hospital of Inner Mongolia Medical University and 15 healthy controls (8 males, 7 females). All participants were between the ages of 58 and 80. PD patients were included if they met the primary PD diagnostic criteria established by the UK Parkinson’s Disease Brain Bank in 1997: (1) a confirmed diagnosis of primary PD; (2) no history of autoimmune diseases; (3) no history of inflammatory diseases; (4) no prior use of medications that could affect dopamine (DA) levels; and (5) Hoehn–Yahr (H–Y) stage 1–1.5 at the time of consultation. Patients with primary tremor, PD syndrome due to cerebrovascular disease, multiple system atrophy, progressive supranuclear palsy, Lewy body dementia, other PD-related syndromes, severe dementia, speech disorders, psychiatric disorders affecting emotional expression, and other serious physical conditions (e.g., malignancies or disabilities) were excluded. All participants provided their written informed consent.

#### 2.1.2 Fecal sample collection, DNA extraction, and sequencing

Fresh fecal samples (5–6 g) were collected from the study participants in the early morning and placed in specially designed fecal microbiota preservation containers. After thorough mixing by shaking, the samples were quickly frozen at −80 °C and transported on dry ice for analysis. DNA extraction from feces was performed using the CTAB method, and the purity and integrity of the DNA were assessed via 1% agarose gel electrophoresis (AGE). DNA quantification was conducted using a Qubit® dsDNA Assay Kit in a Qubit® 2.0 Fluorometer (Life Technologies, CA, United States). A suitable volume of sample was diluted in sterile water to achieve an OD value between 1.8 and 2.0. One microgram of genomic DNA was used to construct the library with an NEBNext® Ultra DNA Library Prep Kit for Illumina (NEB, United States), and DNA fragments of approximately 350 bp were randomly sheared using a Covaris ultrasonic disruptor. The library preparation included end-repair, A-tailing, adapter ligation, purification, and PCR amplification. After library construction, initial quantification was performed using a Qubit 2.0 instrument. The library was then diluted to 2 ng/μl, and its insert size was verified using an Agilent 2100 instrument. If the insert size met expectations, quantitative PCR was employed to precisely quantify the effective concentration of the library (effective concentration > 3 nM), ensuring library quality. Upon passing quality checks, different libraries were pooled according to their effective concentration and the required sequencing depth, and sequencing was performed on the Illumina PE150 platform.

#### 2.1.3 Preprocessing of raw sequencing data and metagenomic assembly

Readfq (V8, https://github.com/cjfields/readfq) was used for preprocessing raw data from the Illumina sequencing platform to obtain clean data for subsequent analysis. The following reads were removed: (a) those with low-quality bases (default quality threshold ≤ 38) exceeding a certain proportion (default length: 40 bp); (b) those with N bases reaching a certain proportion (default length: 10 bp); and (c) those with adapter overlaps exceeding a certain threshold (default length: 15 bp). Considering the possibility of host contamination in samples, the clean data were BLASTed against the host database to filter out reads of host origin. Bowtie2 software (version 2.2.4, http://bowtie-bio.sourceforge.net/bowtie2/index.shtml) was applied using the default setting with the following parameters (Karlsson et al., 2012, 2013; Scher et al., 2013): --end-to-end, --sensitive, -I 200, and -X 400. MEGAHIT software (v1.0.4-beta) was used for assembly analyses of clean data, with the following assembly parameters: --presets meta-large (--end-to-end, --sensitive, -I 200, -X 400) (Karlsson et al., 2013; Nielsen et al., 2014). Scaftigs without N were obtained by breaking the resulting scaffolds from the N junction (Qin et al., 2010; Li et al., 2015).

#### 2.1.4 Bioinformatics and statistical analysis

Linear discriminant analysis effect size (LEfSe) (version 1.0) was employed to analyze differences in taxonomic abundance across groups. Initially, taxa were annotated by group classification, and the nonparametric Kruskal–Wallis test was applied to identify taxa with significant differences in abundance (*p* < 0.05). These differences were further validated using the Wilcoxon rank-sum test, and linear discriminant analysis (LDA) was conducted to estimate the effect size of each taxon, with an LDA score threshold set at >2. The results were visualized via bar plots to obtain an intuitive display of taxa with significant intergroup differences and their relative importance, thereby supporting the biological interpretation of functional disparities among groups. Data processing and analysis were conducted via the R packages tidyverse (version 1.3.2) and microeco (version 0.9.1). Spearman correlation analyses were performed according to taxonomic abundance data to calculate pairwise associations between taxa. Significant and strongly correlated taxa pairs (|r| > 0.6, *p* < 0.05) were identified via the R packages psych (version 2.2.1), reshape2 (version 1.4.4), and igraph (version 1.3.5) and used to construct a co-occurrence network. The network was visualized with Cytoscape (version 3.8.2), and topological parameters such as node degree and clustering coefficient were analyzed to assess synergistic interactions and ecological relationships among microbial taxa, thereby elucidating the complex patterns of microbial community interactions. Additionally, STAMP (version 2.1.3) was used to analyze differences in gene abundance between groups. Gene abundance data were first normalized and grouped, and statistical tests such as Welch’s *t* test or the G test were employed to determine genes with significantly different expression levels. Multiple testing corrections were performed via the Benjamini–Hochberg method (FDR < 0.05) to identify significant genes. The results were visualized via bar plots or scatter plots in STAMP, offering an intuitive display of gene abundance distribution and significance levels across groups, thereby revealing biologically relevant gene-level differences. Finally, Spearman correlation analysis was applied to compute the correlation coefficients and *p* values between the gene and taxonomic abundance profiles. Correlation and significance matrices were generated and visualized as heatmaps using the pheatmap package (version 1.0.12). Correlation strengths are represented by a color gradient (ranging from negative to positive), and significant correlations (*p* < 0.05) are indicated by asterisks (*).

### 2.2 Mendelian randomization

#### 2.2.1 Data sources and selection of instrumental variables

The gut microbiota data were derived from the publicly available MiBioGen consortium dataset, which includes 24 cohorts comprising 18,340 individuals from diverse ancestries (Kurilshikov et al., 2021). After rigorous quality control, taxa with a prevalence < 20% and unclassified groups were excluded, leaving 196 bacterial units (phylum, class, order, family, and genus). Genotyping was performed with a call rate and imputation quality filter, with minor allele frequency exceeding 0.05. A regression model was applied using age, sex, batch, and genetic principal components as covariates. Genome-wide association studies (GWASs) were conducted for each microbial feature, and false discovery rate (FDR) correction was applied for multiple comparisons (Kurilshikov et al., 2021). To ensure the robustness and reliability of Instrumental variables (IVs) in MR analysis, we implemented a series of stringent quality control criteria to select genetic variants significantly associated with gut microbiota traits. Specifically, to obtain a sufficient number of IVs while maintaining significance, we applied a selection threshold of *p* < 1 × 10^−5^. This threshold has been widely adopted in microbiome studies and facilitates the identification of a broad set of IVs with adequate statistical power, thereby providing reliable genetic instruments for subsequent MR analyses (Sanna et al., 2019). To minimize the impact of linkage disequilibrium (LD) on the analysis and to ensure the independence of IVs, we performed LD pruning on the selected single nucleotide polymorphisms (SNPs). Specifically, SNPs with an LD r^2^ < 0.1 within a 500-kb window were removed to reduce redundant information arising from genetic correlations among SNPs (Ni et al., 2022). In addition, we calculated the F statistic for each SNP to assess the strength of the IVs in explaining gut microbiota phenotypes and to mitigate bias arising from weak instruments (Burgess et al., 2011). SNPs with F statistics less than 10 were excluded, and only those with sufficiently large F statistics were retained for subsequent analyses. These high-quality instrumental variables provide a robust genetic basis for causal inference and reduce the risk of potential bias.

The PD GWAS dataset used in this study was sourced from the FinnGen database, which integrates large-scale genomic data from the Finnish Biobank along with corresponding health records. The dataset included 5,861 diagnosed PD patients and 494,487 non-PD control individuals to ensure the robustness and representativeness of the statistical analysis. This GWAS dataset encompasses 21,327,062 SNPs and was filtered using standard quality control procedures to exclude low-quality variants and those potentially introducing bias. Data analyses were conducted as per the FinnGen research protocol, and the relevant GWAS results are available from the official FinnGen database^1^ for further research analysis and validation. To further validate the MR findings, we supplemented our results with GWAS data from the International Parkinson’s Disease Genomics Consortium (IPDGC). This study included 482,730 participants, comprising 33,674 PD patients and 449,056 controls of European descent, covering 17,891,936 SNPs.

#### 2.2.2 Statistical analysis

All calculations were performed using R software (version 4.4.1). The primary causal estimates were obtained using the inverse variance weighted (IVW) method. The IVW method provides the most efficient and unbiased estimates when the SNPs fully satisfy the assumptions of MR; however, if some instrumental variables exhibit horizontal pleiotropy, the IVW method may be affected to some extent, leading to biased causal estimates. Several sensitivity analyses were conducted to complement the primary analysis and assess the robustness of the results. First, we applied the weighted median (WM) method, which can provide reliable causal estimates even when up to 50% of the instrumental variables are invalid. Second, MR–Egger regression was employed to account for the possibility that all instrumental variables may be invalid and to detect horizontal pleiotropy. An intercept term significantly deviating from zero in the MR–Egger regression test indicates that the results may be influenced by pleiotropic bias. Additionally, the MR-pleiotropy residual sum and outlier (MR-PRESSO) method was applied to identify and correct outliers, thereby improving the accuracy of the causal estimates. To evaluate the quality of the instrumental variables, Cochran’s Q statistic was calculated to detect heterogeneity among the SNPs. A Q statistic *p* value less than 0.05 indicates significant heterogeneity between the SNPs. In such cases, random-effects IVW was used for causal inference to mitigate the potential impact of heterogeneity. Finally, a leave-one-out analysis was conducted, where each SNP was sequentially removed, and the changes in the overall effect were observed to assess whether any individual instrumental variable exerted an undue influence on the results.

## 3 Results

### 3.1 Differences in the composition of the gut microbiota

**Figure 1:**
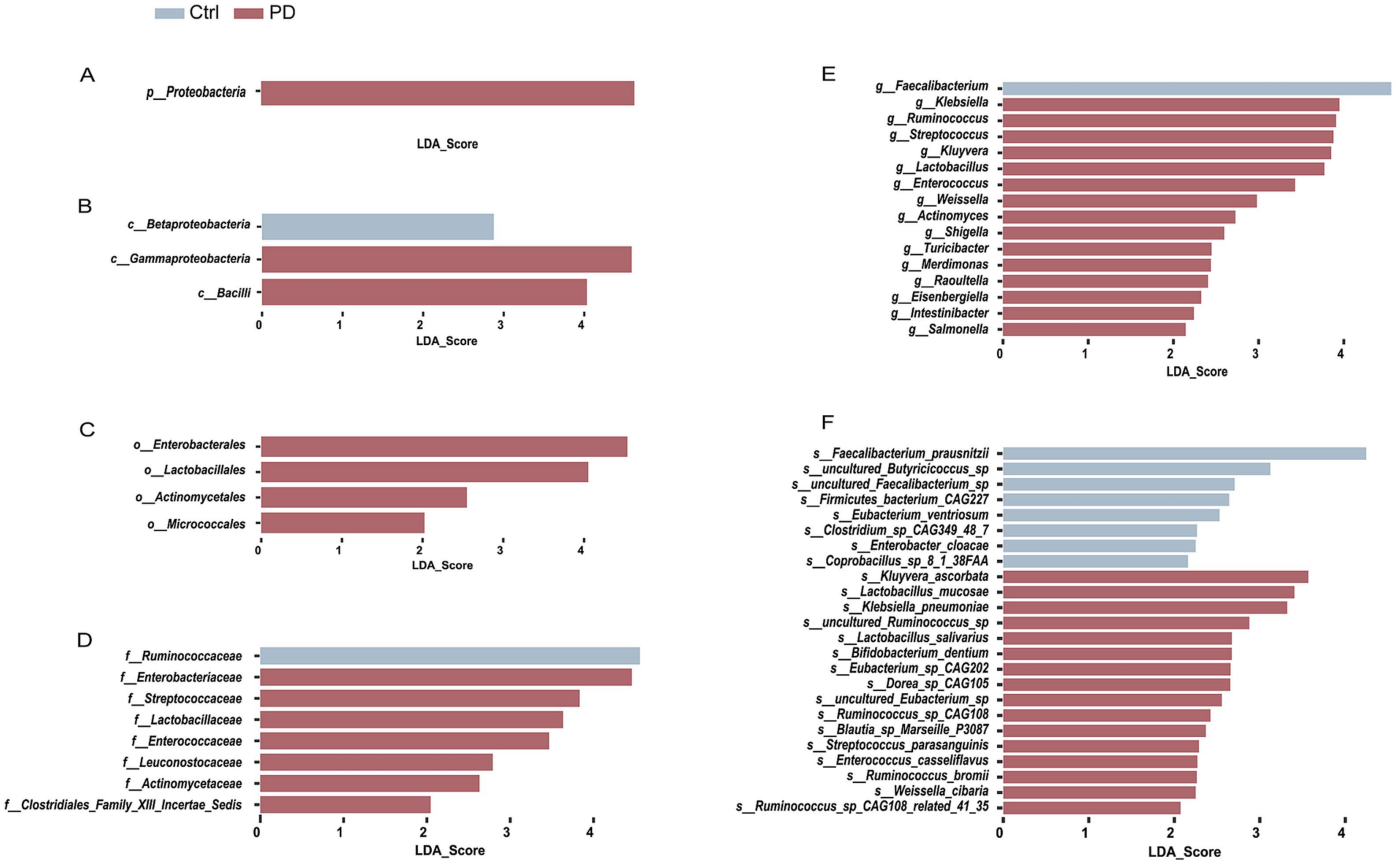
LEfSe analysis revealed differences in the microbiota composition between PD patients and healthy controls. Each bar graph represents the linear discriminant analysis (LDA) score of different microbial taxa at the phylum **(A)**, class **(B)**, order **(C)**, family **(D)**, genus **(E)**, and species **(F)** levels between the two groups. A higher LDA score indicates a greater enrichment of that particular taxon in the corresponding group. The blue bars indicate microbes dominating in the healthy control group, whereas the red bars indicate those dominating in the PD patient group.

To investigate the differences in the gut microbiota composition between patients with PD and healthy individuals, we conducted a differential analysis across multiple taxonomic levels (phylum, class, order, family, genus, and species) using the LEfSe method to identify potential key biological biomarkers. Our results revealed significant taxonomic differences in the overall microbial composition between the PD group and healthy controls. At the phylum level (Figure 1A), *Proteobacteria* was predominant in the PD group (LDA score = 4.50, *p* = 0.037), whereas no dominant phylum was observed in the healthy controls. Further analysis at more refined taxonomic levels revealed that the relative abundances of *Gammaproteobacteria* and *Bacilli* were significantly greater in the PD group than in the healthy group (LDA scores = 4.59, *p* = 0.020 and 4.03, *p* < 0.001, respectively) (Figure 1B). In contrast, *Betaproteobacteria* was more abundant in the healthy control group (LDA score = 2.88, *p* = 0.043).

At the order level (Figure 1C), the abundances of *Enterobacterales* and *Lactobacillales* were significantly enriched in the PD patient group (LDA scores of 4.54, *p* = 0.035, and 4.05, *p* = 0.001, respectively). Additionally, *Actinomycetales* and *Micrococcales* were more prevalent in PD patients (LDA scores of 2.55, *p* < 0.001, and 2.02, *p* < 0.001, respectively), further supporting the structural alterations of the gut microbiota in PD.

At a more refined taxonomic (family) level (Figure 1D), the relative abundances of *Enterobacteriaceae*, *Streptococcaceae*, *Lactobacillaceae*, and *Enterococcaceae* were significantly greater in the PD group than in the healthy control group, with LDA scores of 4.46 (*p* = 0.049), 3.83 (*p* = 0.002), 3.63 (*p* < 0.001), and 3.47 (*p* = 0.037), respectively. In contrast, the family *Ruminococcaceae* was more enriched in the healthy control group (LDA score = 4.55, *p* = 0.040).

At the genus level (Figure 1E), the relative abundances of *Klebsiella*, *Streptococcus*, and *Lactobacillus* were significantly greater in the PD group than in the healthy control group (LDA scores: 3.95, *p* = 0.003; 3.88, *p* = 0.006; and 3.77, *p* < 0.001, respectively). These alterations suggest the potential enrichment of pathogenic taxa in the gut microbiota of PD patients, which may contribute to intestinal inflammation and metabolic dysregulation. In contrast, the genus *Faecalibacterium* was significantly more abundant in the healthy control group (LDA score = 4.56, *p* = 0.043), indicating its possible role in maintaining gut health and exerting anti-inflammatory effects.

Further species-level analysis revealed specific differential bacterial taxa (Figure 1F). In the healthy control group, the abundances of *Faecalibacterium prausnitzii* and *uncultured Butyricicoccus* sp. were significantly greater than those in the PD patients (LDA scores of 4.24, *p* = 0.020; 3.12, *p* = 0.026, respectively). In contrast, the abundance of pathogenic bacteria such as *Klebsiella pneumoniae*, *Streptococcus*, and *Lactobacillus mucosae* was markedly elevated in the PD patient group (LDA scores of 3.32, *p* = 0.003; 2.29, *p* = 0.012; 3.40, *p* = 0.005, respectively).

### 3.2 Analysis of species co-occurrence networks

**Figure 2:**
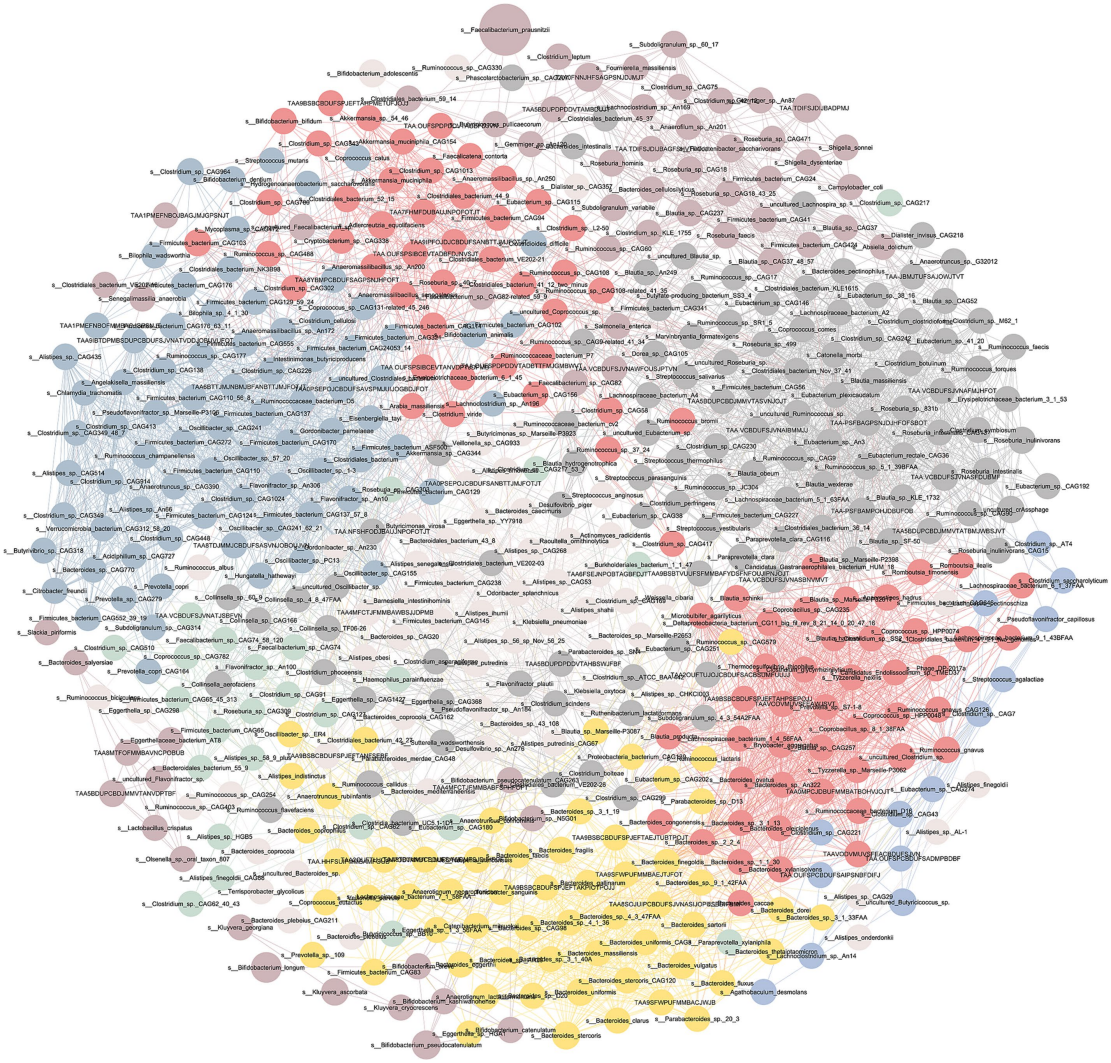
Gut microbiota interaction network in PD. Nodes represent species, whereas edges indicate the correlation between two species. The thickness and color of the edges reflect the strength and direction (positive or negative) of the correlation, respectively.

Our analysis of positive correlations revealed several bacterial strains exhibiting significant synergistic interactions (Figure 2). For example, the correlation coefficient between *Alistipes* and *Clostridium* reached r = 1.000, indicating an almost perfect positive correlation. Similarly, the strong positive correlations within *Alistipes* (r = 1.000) and between *Alistipes* and *Clostridium* (r = 0.999) suggest that these bacteria may coexist in the intestinal environment and potentially collaborate in certain metabolic processes or in maintaining the intestinal ecological balance. Notably, the strong correlations of *Clostridium_*sp.*_CAG349* with *Alistipes_*sp.*_CAG514* (r = 1.000) and *Clostridium_sp._CAG349_48_7* (r = 0.999) further support the possibility of synergistic interactions between these species in the gut. Additionally, *Verrucomicrobia_bacterium_CAG312_58_20* was significantly positively correlated with all of the aforementioned strains (correlation coefficients ranging from approximately 0.999 to 1.000), suggesting that this species of the *Verrucomicrobia* phylum may play a key role in the formation of stable symbiotic networks with other strains. The most notable negative correlations were between *Faecalibacterium_prausnitzii* and *Blautia_schinkii* (r = −0.527), as well as *Eisenbergiella* (r = −0.538).

### 3.3 Analysis of the abundance differences in the functional genes of the gut microbiota

**Figure 3:**
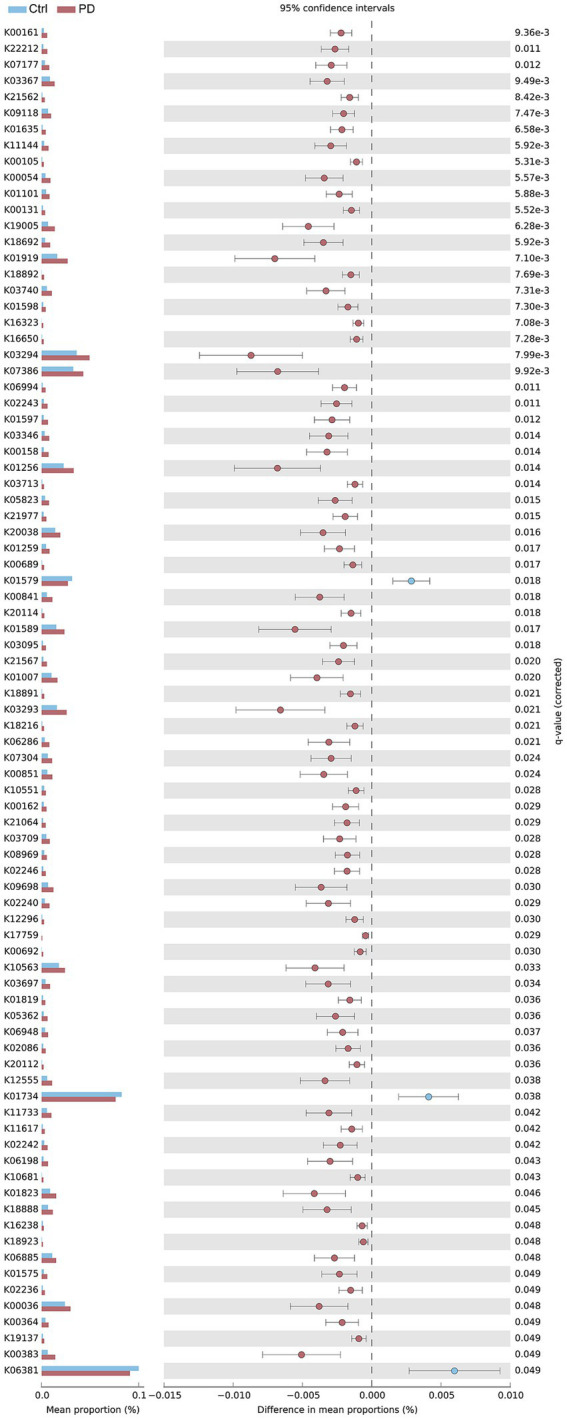
Comparison of gene expression between healthy controls and PD patients. The left bar chart illustrates the expression levels of various genes in both groups, whereas the right dot plot, with error bars, shows the average expression levels of each gene in the two groups along with their variability. Blue represents the healthy control group, and red represents the PD patient group.

We subsequently compared the functional gene abundances of the gut microbiota between the PD patient and healthy control groups. The results revealed significant upregulation of several metabolism-related genes in the PD group (Figure 3), suggesting that the gut microbiota may undergo functional reprogramming in response to inflammation and oxidative stress. First, regarding carbohydrate metabolism and energy pathways, we observed that the abundances of K00105 (alcohol dehydrogenase, EC: 1.1.1.2) and K00131 (glyceraldehyde-3-phosphate dehydrogenase, EC: 1.2.1.9) were significantly greater in the PD patient group than in the healthy control group (*p* < 0.006). Additionally, the levels of K00054 (pyruvate kinase, EC:2.7.1.40) and K01101 (*β*-galactosidase, EC:3.2.1.23) were increased in the PD patient group, suggesting an increase in glucose metabolism-related pathways in these patients. Similarly, the abundance of genes associated with the respiratory chain was also significantly increased in the PD patient group. For example, the expression levels of K18692 (NADH:quinone oxidoreductase subunit 1, EC:1.6.5.3) and K21562 (NADH dehydrogenase, EC:1.6.5.3) were elevated, indicating that the gut microbiota in PD patients may exhibit adaptive changes in energy production. Further analysis of genes related to protein metabolism and homeostasis revealed that the abundances of K19005 (ubiquitin-conjugating enzyme E2 N, EC:2.3.2.23) and K22212 (ubiquitin-specific protease 10, EC:3.4.19.12) were significantly greater in the PD patient group than in the control group, suggesting alterations in the gut microbiota’s function in protein degradation and stability regulation. Moreover, genes associated with nucleic acid metabolism and DNA repair were upregulated in the PD patient group. For example, K11144 (DNA methyltransferase, EC:2.1.1.37) and K03346 (DNA methyltransferase 1, EC:2.1.1.37) expression levels were elevated, suggesting that these genes may play crucial roles in maintaining genomic stability and repairing DNA damage. In terms of amino acid metabolism, the abundances of K01635 (glutamine synthetase, EC:6.3.1.2) and K01598 (adenosine deaminase, EC:3.5.4.4) were increased in the PD patient group, further indicating that changes in amino acid metabolism might have occurred in the gut microbiota of these patients. The elevated abundances of K01256 (*β*-glucosidase, EC: 3.2.1.21) and K01597 (guanine deaminase, EC: 3.5.4.3) support this conclusion. Notably, the increased abundance of K03294 (ribosomal protein L3, EC:3.6.5.3) suggested enhanced protein synthesis and translation functions in the PD patient group, which may reflect the potential role of the microbiota in regulating cellular functions.

### 3.4 Significant differences in functional metabolic pathways

**Figure 4:**
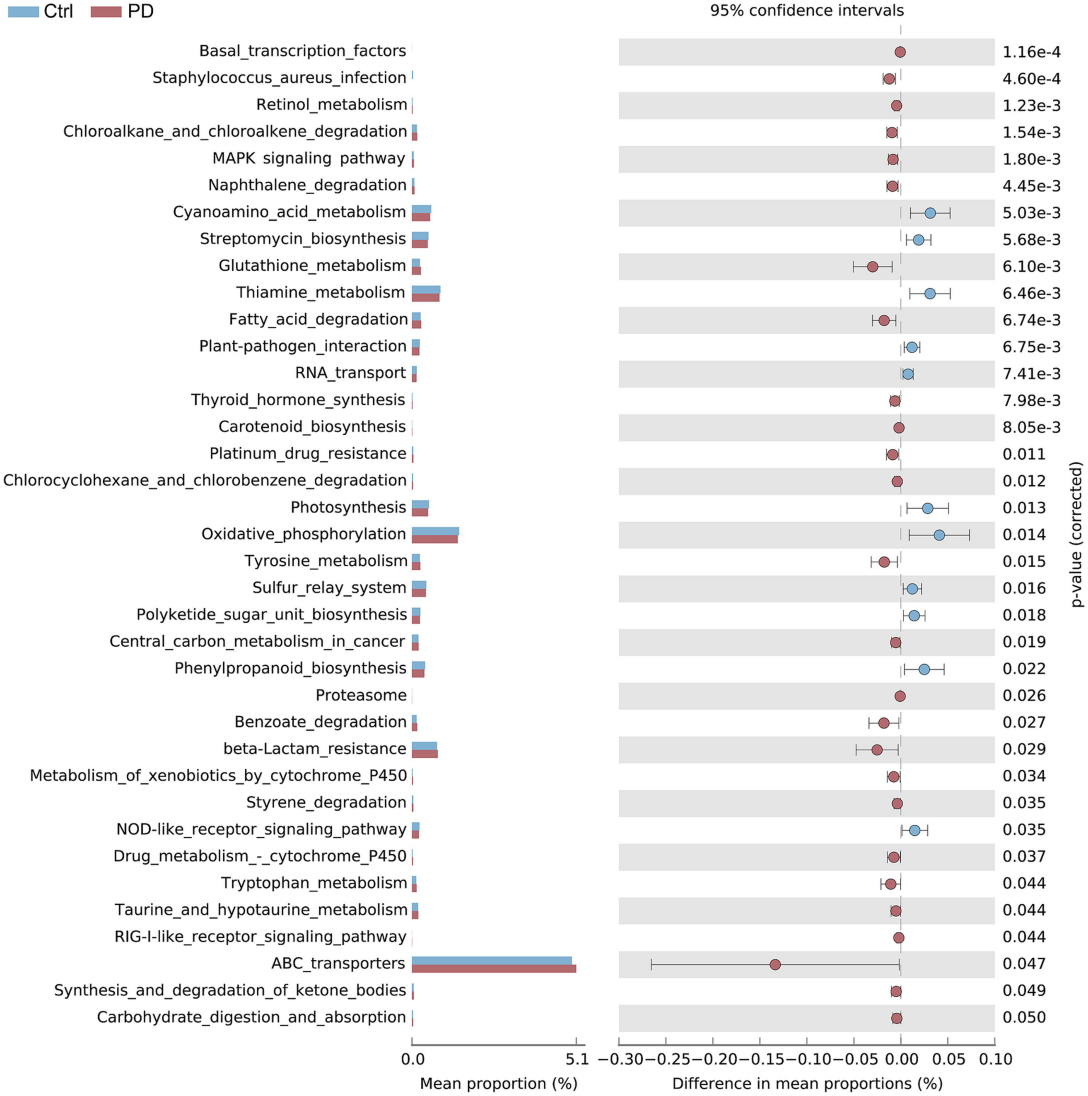
Comparison of metabolic pathway activity between the healthy control group and the PD patient group. The bar charts on the left represent the distribution of activity for each pathway. On the right, a dot plot with error bars illustrates the average activity of each metabolic pathway and its range of variation under the two conditions. Blue represents the healthy control group, whereas red represents the PD patient group.

The results of the KEGG pathway analysis (Figure 4) revealed significant differences in several functional metabolic pathways between the PD patient group and the healthy control group, providing new insights into the potential biological differences between the two groups in terms of metabolic activity, signal transduction, environmental adaptation, and immune regulation. Our analysis revealed that the abundance of basal transcription factors was significantly greater in the PD patient group than in the healthy control group (*p* < 0.001). Moreover, the immune regulation and infection-related *Staphylococcus aureus* infection pathways demonstrated significantly higher abundances in the PD patient group (*p* < 0.001), further suggesting that immune responses in the gut microbiota of PD patients may be under stronger regulation. At the metabolic level, the retinol metabolism pathway was significantly elevated in the PD patient group (*p* = 0.001), indicating that this pathway may play a crucial role in PD pathogenesis. Additionally, the MAPK signaling pathway (plant) and naphthalene degradation pathway were significantly more active in the PD patient group than in the healthy control group (*p* < 0.005), suggesting that these pathways may be closely associated with the pathological processes of PD. Notably, glutathione metabolism was significantly upregulated in the PD patient group (*p* = 0.006), possibly reflecting the metabolic adaptation of patients in response to oxidative stress. Furthermore, the thiamine metabolism and fatty acid degradation pathways were significantly more abundant in the PD patient group (*p* < 0.007), further supporting potential alterations in energy metabolism within the gut microbiota of PD patients. The oxidative phosphorylation and ketone body synthesis and degradation pathways exhibited slight increases in activity in the PD patient group (*p* < 0.05), suggesting that the gut microbiota may play a role in energy metabolism and homeostasis maintenance in PD patients. Finally, the carbohydrate digestion and absorption pathway was also somewhat enriched in the PD patient group (*p* = 0.050), indicating that this metabolic pathway may be involved in gut microbiota regulation in PD.

### 3.5 Correlation heatmap

**Figure 5:**
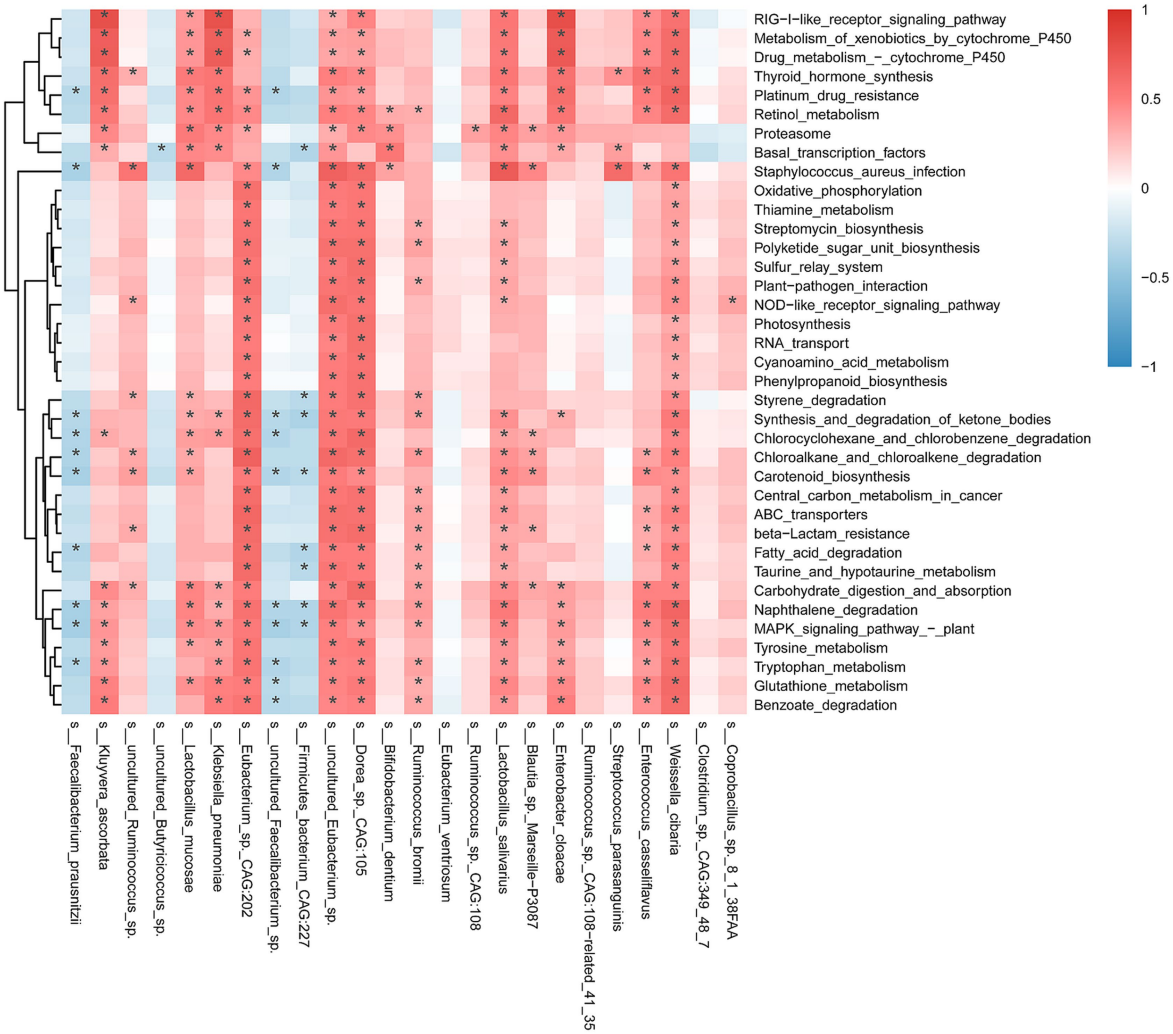
Heatmap of microbiota taxonomic abundance and associated metabolic pathways. The figure shows the magnitude of the correlation coefficients, with red indicating positive correlations and blue indicating negative correlations. Asterisks “*” within each cell indicate statistically significant correlations (*p* < 0.05).

We revealed a complex landscape of interactions between key bacterial species and metabolic pathways within the gut microbiota functional metabolic network of PD patients (Figure 5). Regarding positive correlations, *Eubacterium* sp. *CAG:202* has garnered particular attention. This strain was significantly positively correlated with multiple metabolic pathways, including the ABC transporter pathway (r = 0.646, *p* < 0.0001), oxidative phosphorylation (r = 0.587, *p* < 0.001), thiamine metabolism (r = 0.547, *p* < 0.001), and *β*-lactam resistance (r = 0.615, *p* < 0.0001). In contrast, certain bacteria, widely recognized for their anti-inflammatory properties, exhibited negative correlations with multiple metabolic pathways. *Faecalibacterium prausnitzii* is a representative example that was significantly negatively correlated with glutathione metabolism (r = −0.305, *p* < 0.05), benzoate degradation (r = −0.281, *p* < 0.05), and β-lactam resistance (r = −0.241, *p* < 0.05). *Faecalibacterium prausnitzii* has known anti-inflammatory properties; thus, reduced anti-inflammatory bacteria in an inflammatory environment may promote an imbalance mechanism that overactivates metabolic pathways, thereby exacerbating disease progression. Moreover, pathways closely related to energy metabolism, such as oxidative phosphorylation and fatty acid degradation, were significantly enriched in the PD patient group and were negatively correlated with beneficial microbiota, including *Faecalibacterium prausnitzii* and uncultured *Ruminococcus* sp., further implying that dysbiosis of the microbiota may disrupt intestinal metabolic homeostasis to some extent. The significant negative correlations observed in vitamin-related pathways, such as amine and retinol metabolism, also suggest that these key metabolic pathways may play important roles in gut microbiota metabolic dysregulation in PD.

### 3.6 Genetic and sensitivity analysis of the gut microbiota in PD patients

**Figure 6:**
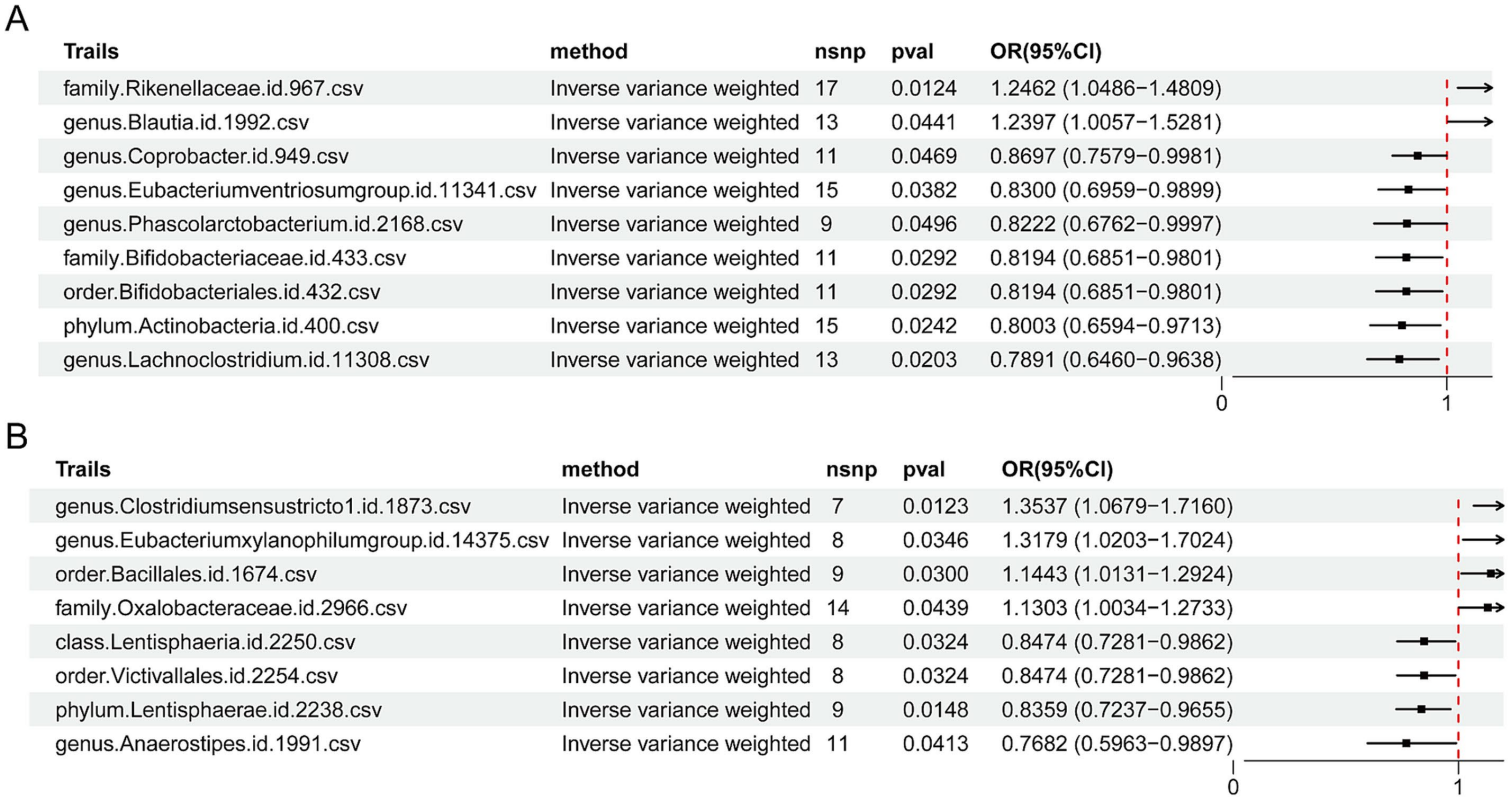
MR analysis suggested causal relationships between the gut microbiota and PD-associated traits. **(A)** Forest plot showing that, as per FinnGen data, the gut microbiota significantly impacts specific PD-related traits. **(B)** Forest plot of IPDGC data indicating that the gut microbiota has a significant effect on PD-related traits.

The IVW method was used to preliminarily explore the causal relationships between gut microbiota characteristics and PD risk. First, using data from the FinnGen database, we identified significant positive associations between the abundance of the Rikenellaceae family (OR = 1.246; 95% CI = 1.049–1.481, *p* = 0.012) and the Blautia genus (OR = 1.240; 95% CI = 1.006–1.528, *p* = 0.044) and PD risk (Figure 6A), suggesting that these microbiota may play a pathogenic role in the development of PD. These findings support the potential involvement of the gut microbiota in neurodegenerative diseases. Moreover, we observed that increased abundances of seven other microbiota, including the Bifidobacteriaceae family (OR = 0.819; 95% CI = 0.685–0.980, *p* = 0.029), were significantly associated with reduced PD risk, indicating a protective role of these microbiota. In particular, the protective effect of Bifidobacteriaceae may act by promoting gut health, enhancing host immune system function, or improving GBA signaling, providing new directions for future experimental research. To further validate these preliminary results, we conducted supplementary analysis using the IPDGC database, enhancing the breadth and consistency of the findings (Figure 6B). In the IPDGC dataset, we detected potential positive causal relationships between the Oxalobacteraceae family (OR = 1.130; 95% CI = 1.003–1.273, *p* = 0.044), Clostridium sensu stricto 1 genus (OR = 1.354; 95% CI = 1.068–1.716, *p* = 0.012), *Eubacterium xylanophilum* group (OR = 1.318; 95% CI = 1.020–1.702, *p* = 0.035), and Bacillales order (OR = 1.144; 95% CI = 1.013–1.293, *p* = 0.030) and PD risk. We also observed several microbiota that were negatively correlated with PD risk at the class or genus level. These included the order Lentisphaeria (OR = 0.836; 95% CI = 0.724–0.965, *p* = 0.015), the genus Anaerostipes (OR = 0.847; 95% CI = 0.728–0.986, *p* = 0.032), and the order Victivallales (OR = 0.847; 95% CI = 0.728–0.986, *p* = 0.032), suggesting that these microbiota may have a protective effect in slowing the progression of PD. Future studies should further investigate whether the negative correlations between these microbiota and PD are related to their functional roles within the gut microbiota community, particularly focusing on how they influence GBA signaling and neuroprotection.

Sensitivity analyses using various methods indicated that the effect sizes reported above are robust (Table 1), with no evidence of pleiotropy or significant heterogeneity (*p* > 0.05). Thus, our findings are reliable, and no other significant associations between the gut microbiota and PD were observed.

**Table 1:** Sensitivity analyses of MR analyses of PD on the gut microbiota by MR–Egger, simple mode, weighted median, weighted mode, and MR–Egger tests for heterogeneity.

Source of outcome data	Exposure level	Microbiota	MR Egger		Simple mode	Weighted median			Weighted mode		Cochran’s Q	
			OR (95%CI)	*p* value	OR (95%CI)	*p* value	OR (95%CI)	*p* value	OR (95%CI)	*p* value	Q	Q*_p* value
FinnGen database	family	Rikenellaceae	1.076 (0.630,1.840)	0.792	1.254 (0.830,1.893)	0.298	1.212 (0.960,1.530)	0.106	1.260 (0.852,1.863)	0.265	13.781	0.542
FinnGen database	genus	Blautia	1.127 (0.649,1.958)	0.680	1.367 (0.852,2.194)	0.219	1.300 (0.988,1.711)	0.061	1.356 (0.861,2.135)	0.214	4.490	0.953
FinnGen database	genus	Coprobacter	0.857 (0.490,1.500)	0.603	0.957 (0.707,1.296)	0.783	0.930 (0.773,1.120)	0.444	0.960 (0.713,1.292)	0.792	9.690	0.376
FinnGen database	genus	Eubacteriumventr-iosumgroup	0.617 (0.280,1.357)	0.251	0.909 (0.585,1.412)	0.677	0.862 (0.686,1.084)	0.203	0.909 (0.611,1.352)	0.644	6.860	0.909
FinnGen database	genus	Phascolarctobact-erium	1.760 (0.705,4.393)	0.265	0.679 (0.450,1.024)	0.102	0.775 (0.602,0.996)	0.047	0.691 (0.466,1.024)	0.103	4.972	0.663
FinnGen database	family	Bifidobacteriaceae	0.685 (0.382,1.230)	0.237	0.922 (0.586,1.450)	0.732	0.868 (0.677,1.112)	0.263	0.925 (0.668,1.279)	0.646	7.171	0.619
FinnGen database	order	Bifidobacteriales	0.685 (0.382,1.230)	0.237	0.922 (0.586,1.450)	0.732	0.868 (0.677,1.112)	0.263	0.925 (0.668,1.279)	0.646	7.171	0.619
FinnGen database	phylum	Actinobacteria	0.824 (0.351,1.934)	0.663	0.841 (0.509,1.390)	0.511	0.843 (0.641,1.107)	0.219	0.841 (0.516,1.370)	0.498	15.073	0.303
FinnGen database	genus	Lachnoclostridi-um	1.284 (0.650,2.534)	0.487	0.708 (0.437,1.149)	0.187	0.744 (0.564,0.982)	0.037	0.705 (0.437,1.139)	0.179	8.673	0.652
IPDGC database	genus	Clostridiumsensu-stricto1	1.728 (1.009,2.959)	0.103	1.404 (0.931,2.116)	0.156	1.413 (1.030,1.940)	0.032	1.416 (0.950,2.110)	0.138	1.614	0.900
IPDGC database	genus	Eubacteriumxyla-nophilumgroup	1.614 (0.708,3.678)	0.298	1.451 (0.798,2.637)	0.261	1.409 (0.991,2.003)	0.056	1.451 (0.856,2.460)	0.209	5.740	0.453
IPDGC database	order	Bacillales	1.133 (0.614,2.092)	0.701	1.215 (0.922,1.601)	0.203	1.179 (0.998,1.393)	0.053	1.221 (0.928,1.606)	0.192	7.820	0.349
IPDGC database	family	Oxalobacteraceae	1.422 (0.856,2.362)	0.198	1.194 (0.913,1.561)	0.217	1.177 (1.005,1.379)	0.043	1.202 (0.942,1.532)	0.162	5.427	0.942
IPDGC database	class	Lentisphaeria	0.743 (0.450,1.225)	0.288	0.747 (0.529,1.056)	0.143	0.783 (0.640,0.959)	0.018	0.751 (0.544,1.036)	0.125	5.789	0.447
IPDGC database	order	Victivallales	0.743 (0.450,1.225)	0.288	0.747 (0.529,1.056)	0.143	0.783 (0.640,0.959)	0.018	0.751 (0.544,1.036)	0.125	5.789	0.447
IPDGC database	phylum	Lentisphaerae	0.715 (0.431,1.186)	0.235	0.743 (0.553, 0.999)	0.085	0.762 (0.628,0.924)	0.006	0.745 (0.565,0.982)	0.070	5.899	0.552
IPDGC database	genus	Anaerostipes	0.568 (0.254,1.270)	0.201	0.976 (0.538,1.769)	0.938	0.792 (0.560,1.120)	0.187	1.024 (0.557,1.884)	0.940	4.561	0.871

## 4 Discussion

The introduction of the GBA theory has expanded the research perspective on the pathogenesis of PD, shifting the focus from the traditional view of central neurodegeneration to the regulatory role of the gut microbiota (Cryan et al., 2019). In this study, we combined metagenomic analysis with MR to systematically explore the genetic associations between the gut microbiota and PD, providing new insights into their potential causal relationships. Several microbial taxa and associated functional pathways were significantly altered in PD patients. Furthermore, a series of microbial taxa significantly associated with PD risk were identified in two independent databases (FinnGen and IPDGC). Some of these taxa exhibited consistent disease-promoting or protective trends, strengthening the robustness and reliability of the findings.

First, from the perspective of microbiome composition, the gut microbiota of PD patients exhibits structural remodeling, characterized particularly by a significant reduction in anti-inflammatory bacterial populations. Notably, a decrease was observed in well-known SCFA-producing bacteria, such as *Faecalibacterium* and members of the *Ruminococcaceae* family; these bacteria are involved in regulating immune responses, intestinal inflammation, and the abnormal aggregation of *α*-Syn (Zhuang et al., 2019; Soto-Martin et al., 2020; Elford et al., 2024). Moreover, in MR analyses, SCFA-producing bacteria such as *Lachnoclostridium* and *Eubacterium ventriosum* are often negatively correlated with PD risk (Zhang et al., 2020; Palepu et al., 2024). SCFAs serve as the primary energy source for colonic epithelial cells and play crucial roles in immunomodulation and anti-inflammatory responses in PD. These effects are mediated through mechanisms such as maintaining intestinal barrier integrity, suppressing the expression of proinflammatory cytokines, and inducing regulatory T (Treg) cell differentiation (van der Hee and Wells, 2021; Blaak et al., 2020; Zhou et al., 2018; Li et al., 2021). In contrast, a significant increase in the gut microbiota is associated with inflammation and metabolic disorders in PD patients (Aho et al., 2021; Pfaffinger et al., 2025). For example, pathogenic genera such as *Klebsiella pneumoniae* and *Streptococcus*, which are notably enriched in PD patients, can disrupt the intestinal barrier, increase permeability, and facilitate the penetration of toxins that, in turn, activate systemic immune responses and induce neuroinflammation (Tazi et al., 2010; Wang et al., 2024). However, the MR analysis in this study did not reveal any significant genetic associations between these harmful bacterial taxa and PD. This finding reinforces the reliability of our metagenomic findings, emphasizing that correlation does not imply causation. This distinction helps prevent overinterpretation and highlights MR’s complementary value in validating microbiome–disease associations, especially in complex diseases like PD. Notably, certain beneficial microbes, such as *Bifidobacterium dentium*, demonstrated a significant protective effect against PD in the MR analyses despite their increased abundance in PD patients. This paradoxical increase may reflect compensatory proliferation, indicating that the SCFAs produced by these bacteria may no longer be sufficient to maintain normal intestinal function (Engevik et al., 2020). Moreover, observational studies have linked elevated levels of *Bifidobacterium* in patients with PD to the dosage of levodopa administered to these individuals (Wallen et al., 2020). Interestingly, however, Blautia and Clostridium—both SCFA-producing bacteria—have demonstrated potential pathogenic roles in MR analyses (Song et al., 2024; Dicks, 2024; Rivera-Chávez et al., 2016). This observation aligns with the findings of an animal study conducted by Qiao et al. (2020), which demonstrated that SCFAs exacerbate the pathological features of PD. These insights offer a more precise microbiota-based intervention strategy for the therapeutic application of probiotics in PD.

Through co-occurrence network analysis, we subsequently identified several microbial pairs exhibiting strong correlations in patients with PD, with SCFA-producing bacteria showing a particularly prominent presence. Notably, strains belonging to the genera *Alistipes* and *Clostridium* demonstrated exceptionally high positive correlations (r = 0.999–1.000), suggesting potentially tight synergistic interactions between these taxa. Considering that both bacterial taxa possess the potential for SCFA synthesis, particularly playing crucial roles in the metabolic pathways of butyrate and propionate, their co-enrichment may represent a compensatory regulatory mechanism by which the host maintains an anti-inflammatory microbial ecology under pathological conditions. This symbiotic pattern may not only modulate local pH and inhibit colonization by pathogenic bacteria but also exert protective effects in maintaining intestinal mucosal integrity and immune homeostasis (Lin et al., 2025; Zhao et al., 2023; Cazzaniga et al., 2024). Thus, we hypothesize that the *Alistipes–Clostridium* symbiotic network may represent a promising target for future microecological interventions in PD, with potential clinical applicability. In contrast, we observed a moderate negative correlation between *Faecalibacterium prausnitzii* and both *Blautia schinkii* and *Eisenbergiella tayi* (r ≈ −0.527 to −0.538). Previous studies have demonstrated that *Faecalibacterium prausnitzii*, a key butyrate-producing bacterium, exerts notable anti-inflammatory and immunomodulatory effects by suppressing proinflammatory cytokines and promoting the induction of Treg cells, thereby significantly alleviating PD-associated intestinal inflammation (Krueger et al., 2025; Bhutta et al., 2024). We hypothesize that the enrichment of *Blautia schinkii* and *Eisenbergiella tayi* may hinder the colonization and functional expression of *Faecalibacterium prausnitzii* through competitive inhibition or interactions involving metabolic products, thereby accelerating the shift of the gut ecosystem from a homeostatic state to a proinflammatory state. This negative relationship may act as a “critical negative regulatory factor” in the process of microbial dysbiosis associated with PD.

By integrating functional gene analysis with KEGG metabolic pathway analysis, we comprehensively investigated the metabolic reprogramming and immune regulation of the gut microbiota in patients with PD, thereby elucidating the potential roles of the microbiota in PD pathogenesis. The increased abundance of metabolic enzyme-encoding genes of the gut microbiota in PD patients, such as alcohol dehydrogenase (K00105), glyceraldehyde-3-phosphate dehydrogenase (K00131), and pyruvate kinase (K00054), suggests that the gut microbiota of PD patients has a markedly increased capacity for glycolysis and energy metabolism pathways, reflecting adaptive responses to altered energy demands during disease progression (Li et al., 2024; Shen et al., 2025; Steiner, 2019). This trend of increased metabolism is consistent with the increased activity observed in the oxidative phosphorylation and carbohydrate digestion and absorption pathways identified through KEGG metabolic pathway analysis, further indicating that the gut microbiota plays a significant role in the metabolic adaptation associated with PD, thereby contributing to the maintenance of energy homeostasis. The most notable finding is the increased abundance of *β*-glucosidase (K01256) in PD patients, suggesting a potential increase in SCFA production within the gut. By promoting SCFA synthesis, the gut microbiota may influence the onset and progression of PD through mechanisms such as the modulation of immune responses, reinforcement of intestinal barrier integrity, and neuroprotection (Śliżewska et al., 2023; Malinowska et al., 2023). This finding corresponds with the significant upregulation of the glutathione metabolism pathway and the *Staphylococcus aureus* infection pathway—both involved in immune regulation—and the marked enhancement of the fatty acid degradation pathway related to SCFA synthesis observed in the PD patient group (Liu et al., 2025; Qin et al., 2024; Ostrakhovitch et al., 2025).

We subsequently investigated the associations between key microbial strains and metabolic pathways to uncover potential regulatory mechanisms and their clinical implications. *Eubacterium_sp._CAG:202* exhibited strong positive correlations with several core metabolic pathways, including energy metabolism, vitamin metabolism, and stress response, suggesting that this strain may play a pivotal role as a “metabolic activator” in the gut of patients with PD. This pathway’s strong correlation with the oxidative phosphorylation and thiamine metabolism pathways indicates its potential involvement in the regulation of gut–brain axis homeostasis through the modulation of energy supply and metabolism associated with neuronal maintenance (Bedarf et al., 2017). This regulatory mechanism may possess neuroprotective significance in the context of impaired energy metabolism in the nervous system of PD patients. Moreover, the association of this mechanism with resistance-related pathways may reflect an adaptive capacity to inflammatory or antibiotic-induced stress conditions, thereby providing microecological support for the stability of the gut microbiota in patients with PD (Zhang et al., 2024). In contrast, *Faecalibacterium prausnitzii* negatively correlated with multiple metabolic pathways, particularly those involved in stress response mechanisms, such as antioxidant activity and aromatic compound metabolism, reflecting a diminished capacity for functional regulation under inflammatory conditions. As a key anti-inflammatory bacterium (Mohebali et al., 2023), reduced *Faecalibacterium prausnitzii* abundance may lead to dysregulation of critical metabolic pathways, thereby triggering compensatory stress responses in the host, such as excessive activation of the glutathione pathway. Overall, our correlation analysis revealed an important trend: the enhancement of microbial metabolic pathways in patients with PD does not necessarily indicate functional optimization but is more likely to represent a stress-induced compensatory response triggered by microbial dysbiosis. The reduction in probiotics exacerbates the negative regulation of metabolic networks, leading to hyperactivation of pathways related to energy production, detoxification, and immune responses, thereby reflecting dysregulation of the regulatory capacity of the microecosystem under disease conditions. This mechanistic insight deepens our understanding of functional imbalances in the PD-associated microbiota and provides a theoretical basis for identifying “keystone strains” with potential regulatory functions.

Despite the insights gained from this study, this study is limited by a small sample size, residual confounding from uncontrolled factors like diet and body mass index (BMI), and population stratification across cohorts. These factors may bias associations between the microbiota and PD, highlighting the need for cautious interpretation and more robust. Future studies should employ larger, ethnically diverse, and longitudinal cohorts to validate these observations and improve generalizability. Incorporating detailed phenotypic data—such as dietary habits, BMI, medication use, and disease-related symptoms—will be essential for more precise adjustment of confounding variables. Furthermore, mechanistic studies are warranted to elucidate the dual and context-dependent roles of SCFA-producing bacteria, which may exert both protective and proinflammatory effects under different physiological conditions (Qiao et al., 2020; Hirayama and Ohno, 2021). Such efforts will be critical to refining microbiome-targeted therapeutic strategies for PD.

## 5 Conclusion

In this work, we provide a novel perspective for exploring the relationship between PD and the gut microbiome. By integrating metagenomics with MR, we found potentially pathogenic commensal bacteria among those traditionally regarded as beneficial, thereby challenging established paradigms in previous research. This finding contributes to a deeper understanding of the clinical relevance of the gut microbiota in PD. Moreover, by revealing alterations in microbial metabolic pathways and their associations with immune responses in PD patients, we highlighted the critical role of SCFA-producing bacteria, indicating a promising direction for microbiota-targeted interventions in PD.

## Data Availability

The raw sequencing data supporting the findings of this study have been deposited in the NCBI Sequence Read Archive (SRA) under the BioProject accession number PRJNA1329258 (https://www.ncbi.nlm.nih.gov/bioproject/PRJNA1329258). These data are publicly available and can be accessed through the NCBI SRA database. The Mendelian randomisation analyses conducted in this study utilise publicly available datasets, accessible at the following addresses: https://r12.finngen.fi/pheno/G6_PARKINSON and https://gwas.mrcieu.ac.uk/datasets/ieu-b-7.

## Footnotes

^1^ https://r12.finngen.fi/pheno/G6_PARKINSON
